# Synovial Sarcoma of the Larynx: Report of a Case and Review of Literature

**DOI:** 10.1155/2017/6134845

**Published:** 2017-02-09

**Authors:** Geetha Narayanan, Anto Baby, Thara Somanathan, Sreedevi Konoth

**Affiliations:** ^1^Department of Medical Oncology, Regional Cancer Centre, Trivandrum 695011, India; ^2^St. Gregorios Medical Mission Hospital, Parumala, Pathanamthitta 689626, India; ^3^Department of Pathology, Regional Cancer Centre, Trivandrum 695011, India; ^4^Department of Radiology, Lourdes Hospital, Kochi 682012, India

## Abstract

Sarcomas account for less than 1% of malignant neoplasms arising in the head and neck in adults. Laryngeal synovial sarcoma is an extremely rare form of laryngeal malignancy with less than 20 cases reported in the literature. We report the case of a 48-year-old man with synovial sarcoma of the larynx. He underwent excision of the tumor followed by radiation. He is alive in remission at 36 months. The literature on synovial sarcoma of the larynx is reviewed.

## 1. Introduction

Sarcomas account for less than 1% of malignant neoplasms arising in the head and neck in adults and less than 5% of soft tissue sarcomas in adults occur in the head and neck region [1–3]. Larynx is a rare primary site in which sarcomas comprise less than 1% of all laryngeal tumors [1]. Synovial sarcoma likewise represents a rare sarcoma histology of which only 3% arise in the head and neck [4]. We report the case of a young man with synovial sarcoma of the larynx.

## 2. Case Report

**Figure 1:**
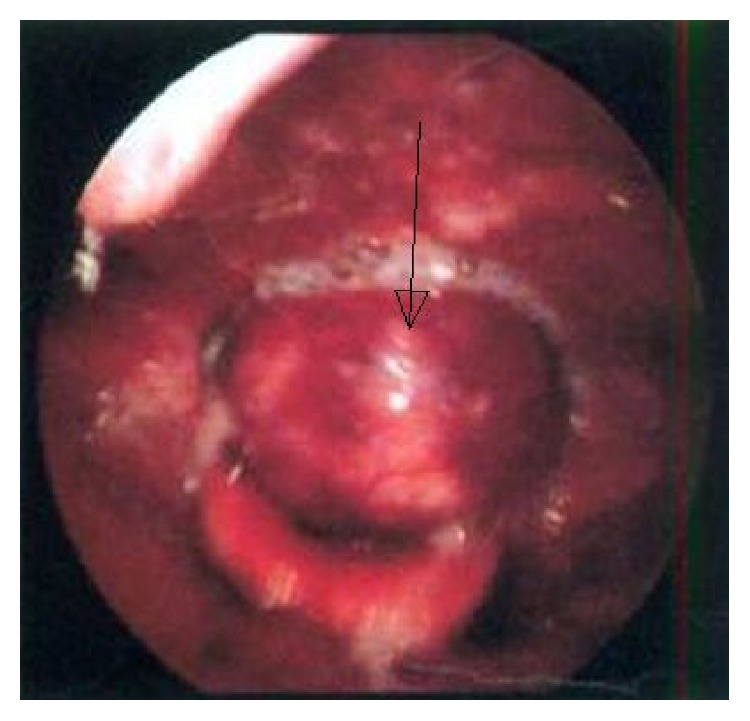
Laryngoscopy showing a pedunculated mass coming from laryngeal inlet.

**Figure 2:**
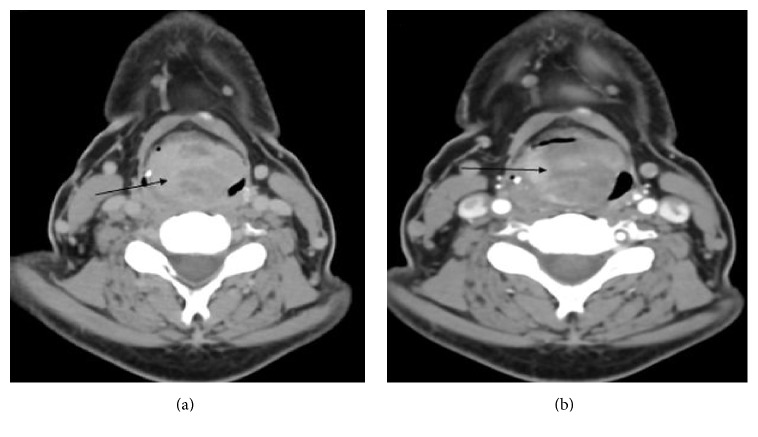
(a) Plain CT scan of the neck showing soft tissue mass at the region of epiglottis with tiny specks of calcifications in the periphery. (b) Postcontrast image showing heterogenous enhancement.

**Figure 3:**
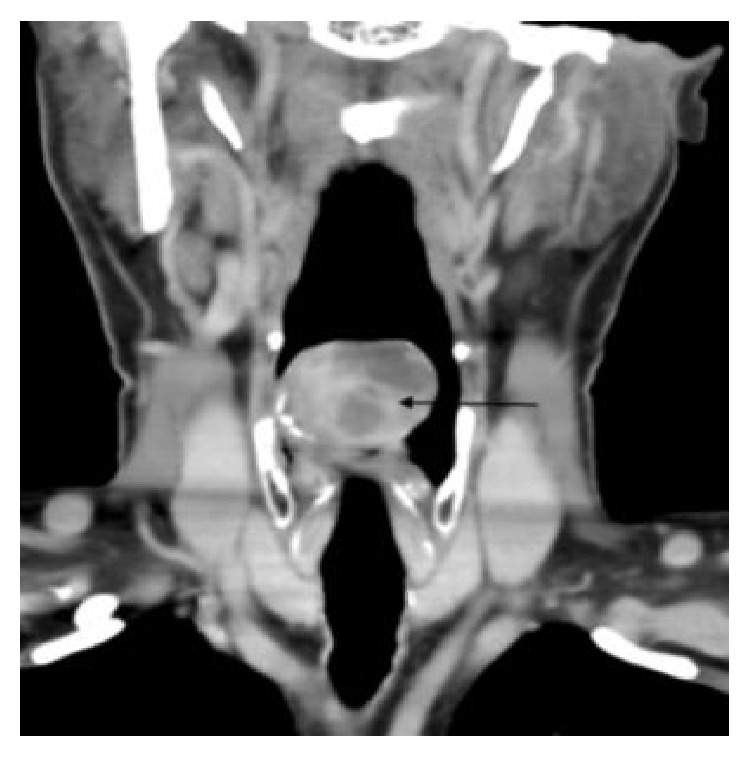
Coronal postcontrast image showing the mass lesion with nonenhancing areas.

**Figure 4:**
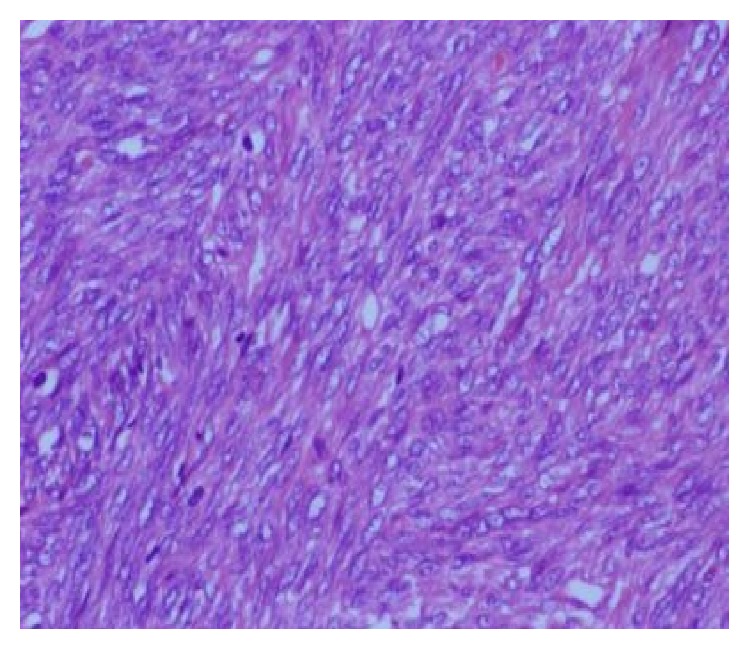
H&E ×40 showing spindle cell sarcoma with hemangiopericytic pattern.

**Figure 5:**
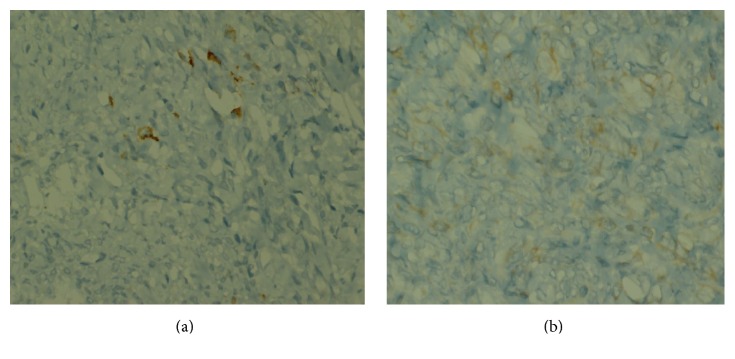
(a) Section showing focal positivity for cytokeratin. (b) Section showing focal positivity for Bcl 2.

A 48-year-old man presented with a history of hoarseness of voice since 5 months, dyspnea since 3 months, and dysphagia since 2 months. He consulted an ENT surgeon where a fibre optic laryngoscopy showed a pedunculated mass occupying the laryngeal inlet and extending up to base of tongue (Figure 1). A computed tomogram (CT) showed a well-defined heterogeneously enhancing lesion, 2.7 × 2.0 × 2.6 cm in the laryngeal inlet in the supraglottic compartment, almost filling it (Figures 2(a), 2(b), and 3). Small cervical lymph nodes were present bilaterally. Patient underwent excision of the mass and tracheostomy. Intraoperatively, a globular pedunculated mass attached to posterior pharyngeal wall and arytenoids was seen. Histopathology examination of the biopsy specimen showed spindle cell sarcoma with hemangiopericytic pattern of intermediate grade, which on immunohistochemistry (IHC) was focally positive for cytokeratin and Bcl 2 (Figures 4, 5(a), and 5(b)). A diagnosis of synovial sarcoma was made. His haemogram, serum chemistries, and CT scan of chest were normal. He was given postoperative external beam irradiation 60 Gy/30#. No chemotherapy was given since it was an intermediate grade tumor. The patient is alive in remission at 36 months.

## 3. Discussion

Laryngeal synovial sarcoma is an extremely rare form of laryngeal malignancy with less than 20 cases reported in the literature (Table 1 [5–23]). Synovial sarcoma is an aggressive malignant soft tissue tumor that is thought to arise from pluripotent mesenchymal cells and usually involve the lower extremities [24]. Sarcomas occur uncommonly in the head and neck region in adults and synovial sarcoma is extremely rare in this site. Squamous cell carcinoma accounts for over 90% of all laryngeal cancers [24]. The median age of patients at diagnosis of the disease is the third decade of life and there is male predominance [25].

Sarcoma of the head and neck commonly presents as painless submucosal or subcutaneous mass and symptoms vary according to the location. Similar to primary squamous cell carcinomas of the larynx, sarcomas in this location produce symptoms secondary to mechanical interference with function dependent on the size. Hoarseness is usually the first symptom; stridor and dyspnea occur subsequently. Dysphagia usually occurs only when the tumor becomes large enough and protrudes into the hypopharynx [26]. In majority of the reported cases the gross appearance has been polypoidal and sometimes infiltrative. Ulceration is rare, in contrast with the early ulceration commonly present in carcinoma of the larynx. Sarcomas may originate in any part of the larynx but most often involve the vocal cords [3, 26]. Our patient also had a similar presentation.

Synovial sarcoma acquired its name due to its microscopic resemblance to developing synovium but is immunophenotypically and ultrastructurally distinct from normal synovium, only rarely arising in joint cavities, and usually occurs in association with para-articular regions of the extremities, with no relation to synovial structures [27]. Histologically, the 2 predominant forms are biphasic and monophasic forms. A branching hemangiopericytoma-like vascular pattern is characteristic and a common finding in both types is the presence of stromal calcification, which ranges from focal to extensive and is an important diagnostic clue [28]. Immunohistochemically, synovial sarcoma is characterised by coexpression of mesenchymal and epithelial markers (cytokeratins and epithelial membrane antigen) [11]. 90% of synovial sarcomas harbour a specific translocation between the SYT gene on chromosome 18 and either the SSX1 or SSX2 gene on the X chromosome [24]. The type of fusion product correlates with the histological pattern; those with SYT-SSX1 are usually biphasic and those with SYT-SSX2 are monophasic. Genetic testing is particularly useful in the poorly differentiated tumors. Our case was positive for cytokeratin and Bcl 2.

The optimal treatment of synovial sarcoma is multimodal. The treatment for sarcoma of the larynx depends on its size, location, and grade. Radical surgical excision is generally accepted as the mainstay of therapy. Because most of the patients can be diagnosed early, conservative surgery with laryngeal preservation is usually possible. Radiotherapy (RT) is an important adjunct in the treatment of soft tissue sarcoma to diminish the incidence of local recurrence [29]. The major indications for postoperative RT are high grade lesions, positive surgical margins, larger tumor (>5 cm), and recurrent lesions [30]. Our patient received postoperative RT.

Adjuvant chemotherapy has been utilized for high grade synovial sarcoma. Doxorubicin and ifosfamide have been shown to demonstrate improvement in disease specific survival in the treatment of soft tissue sarcomas [31, 32]. Our patient was not given chemotherapy since it was an intermediate grade lesion. Disease recurrence is a significant problem, with up to 45% of patients with head and neck synovial sarcoma developing a local recurrence and 33% developing distant metastatic disease [33]. Liu et al. in their review on treatment results of sarcoma of the larynx reported that the 5-year OS of patients with soft tissue sarcoma of the head and neck ranged from 32% to 75% [34]. Our literature review on 20 patients with synovial sarcoma of larynx shows that the survival of these patents ranged from 3 months to 16 years, with 50% alive at 2 years.

In summary, synovial sarcoma of larynx is a rare entity. Surgery is the mainstay of treatment with conservative surgery and organ preservation considered for early cases. Postoperative radiation is reserved for those with positive margins and high grade tumors and chemotherapy for high grade tumors.

## Figures and Tables

**Table 1:** Review of literature of synovial sarcoma of larynx.

Sl.lno	Reference	Age/sex	Site	Treatment	Survival status
1	[5]	28 F	Left hemilarynx hypopharynx	Pharyngolaryngectomy, 1 year lung metastasis, XRT + chemo	2.5 years DIED

2	[6]	23 F	Interarytenoid and left arytenoid area	Supraglottic laryngectomy and total laryngopharyngectomy	12 years NED

3	[7]	76 M	Rt supraglottic area	Laryngectomy	3 years NED

4	[8]	28 F	Rt aryepiglottic fold	Tumorectomy + XRT	3 years NED

5	[9]	28 M	Supraglottis	Supraglottic laryngectomy + RtND + XRT	16 years NED

6	[10]	14 M	Left arytenoid	Excision tumor, local recurrence at 3 years, total laryngectomy + CT + XRT	10 m NED

7	[11]	27 M	Supraglottis	Surgery, local recurrence 3 m, CT + XRT, 3 m hemilaryngectomy	9 m NED

8	[12]	68 F	Cricoids	Laryngopharyngectomy + neck dissection, cervical oesophagectomy	NA

9	[13]	24 M	Supraglottis	Hemilaryngectomy, recurrence at 12 m, total laryngectomy + XRT, lung mets, chemotherapy	42 m NED

10	[14]	16 M	Supraglottis	CO_2_ laser surgery + XRT	24 m NED

11	[15]	54 M	Supraglottis	Laryngopharyngectomy + modified neck dissection	24 m NED

12	[16]	NA	Rt aryepiglottic fold	CO_2_ laser surgery	36 m NED

13	[17]	26 M	Supraglottis	CO_2_ laser surgery	NA

14	[18]	79 M	Supraglottis	Total laryngectomy	3 m NED

15	[19]	57 M	Supraglottis	CO_2_ laser surgery	14 m NED

16	[20]	12 M	Supraglottis	Surgery + chemo	4 m NED

17	[21]	26 M	Larynx	Surgery + XRT	20 m NED

18	[22]	37 M	Supraglottis	Surgery + XRT + chemo	41 m DIED

19	[23]	20 M	Supraglottic larynx	Total laryngectomy, left hemithyroidectomy, left modified radical neck dissection + XRT + chemotherapy	18 m NED

20	Present case	48 M	Supraglottic larynx	Surgery + XRT	36 m NED
